# Larger infarct size associated with dysglycemia at the time of ST-elevation myocardial infarction is related to later presentation

**DOI:** 10.1186/1532-429X-15-S1-O77

**Published:** 2013-01-30

**Authors:** Naveed Razvi, Ben Grundy, Leong Ng, Gerry P McCann, Iain Squire

**Affiliations:** 1 Cardiovascular Sciences, University of Leicester, Leicester, UK; 2 Cardiovascular Biomedical Research Unit, National Institute of Health Research (NIHR), Leicester, UK

## Background

Patients with dysglycemia at the time of ST-elevation myocardial infarction (STEMI) have a worse prognosis. The reasons for this are not entirely clear. Admission hyperglycemia has been associated with larger infarct size.

The aims of this study were to examine the relation of acute and chronic glycemic state to myocardial scar and salvage characteristics in patients with reperfused ST-elevation myocardial infarction.

## Methods

56 patients treated for first ST-elevation myocardial infarction (STEMI) without a known diagnosis of Diabetes mellitus were prospectively enrolled between January and December 2010. Glycosylated hemoglobin (HbA1c) and glucose were sampled on admission to the emergency cardiac ward. Patients underwent CMR during the index admission (median day 2, IQR 2days), with assessment of area-at-risk (STIRs), Infarct size%, late microvascular obstruction, and left ventricular function. Population characteristics are presented in Table 1.

**Table 1:** Patient characteristics

Age (years)	60.77 +/- 11.75
Male	50/56 (89%)

Peak CK (U/l)	1318.8 +/- 1282

Hypertension	20/56 (36%)

Smoking	28/56 (50%)

Hypercholesterolemia	15/56 (27%)

Family history	17/56 (30%)

Diabetes Mellitus	0/56 (0%)

Reperfusion therapy: PPCI thrombolysis	45 (80%) 11 (20%)

Time to reperfusion (minutes) all patients PPCI only thrombolysis	194.16 +/- 121.12 (140) 189.36 +/- 113.86 (120) 213.82 +/- 152.04 (225)

Multivessel disease	22/56 (39%)

LAD culprit	25/56(45%)

Glucose (mmol/l)	7.97 +/- 2.54 (median 7.7, IQR 2.10)

HbA1c (%)	6.67 +- 1.07 (median 5.9, IQR 2.10)

IS% by FWHM	23.08 +/- 12.97 (median 20.31, IQR 16.68)

MVO%	2.02 +/- 3.67 (median 0.49, IQR 3.01)

AAR%	41.19 +/- 13.78 (median 38.78, IQR 22.21)

MSI%	53.71 +/- 26.41 (median 58.96, IQR 35.09)

ECG resolution%	62% +/- 34% (median 67%)

PPCI= Primary Percutaneous Coronary Intervention, LAD= Left Anterior Descending Coronary Artery, IS=Infarct size, MVO=microvascular obstruction, IMH=intramyocardial hemorrhage, AAR=Area at risk, MSI= Myocardial Salvage Index.

**Table 2:** Correlations between infarct characteristics and glucose characteristics:

	Glucose	HbA1c
Peak CK	R=0.152 P=0.267	R=0.120 P=0.393

ECG resolution %	R=-0.393 P=0.003	R=-0.352 P=0.010

Time to reperfusion	R=0.185 P=0.177	R=0.334 P=0.015

Age	R=0.361 P=0.007	R=0.184 P=0.188

IS%	R=0.296 P=0.028	R=0.152 P=0.277

MVO%	R=0.259 P=0.056	R=0.199 P=0.152

IMH%	R=0.187 P=0.176	R=0.292 P=0.036

AAR%	R=0.128 P=0.358	R=-0.176 P=0.213

MSI%	R=-0.187 P=0.176	R=-0.399 P=0.003

IS=Infarct size, MVO=microvascular obstruction, IMH=intramyocardial hemorrhage, AAR=Area at risk, MSI= Myocardial Salvage Index.

Patients with below and above median glucose were compared. Spearman's rank correlation was used to compare non-parametric data. Correlations with a p<0.1 were entered into a multivariate linear regression model. Independent t-tests were used to compare groups above and below the median for glucose and HbA1c levels. There were no significant differences between patients receiving PPCI or thrombolysis, so these were grouped for analysis.

## Results

When patients were dichotomised into glucose levels below (<7.8) and above the median (≥7.8), the supra-median group were significantly older (64.7 years vs. 57.1 years, p=0.018) and had greater infarct size (28.33% vs. 18.46%, p=0.007).

Dichotomising patients by HbA1c into levels below the median (<5.9%) and above the median (≥5.9%), the supra-median group had significantly greater glucose levels (8.8 mmol/l vs. 7.1mmol/l, p=0.010), lower ECG resolution (51.6% vs. 75.5%, p=0.018), greater MVO% (2.77% vs. 1.11%, p=0.049), greater IMH% (1.96% vs. 0.61%, =-0.015), and lower myocardial salvage index (43.30% vs. 65.15%, p=0.003).

On multivariate linear regression analysis however, glucose was not a predictor of IS% (R=0.549, R2=0.302, Age t=3.441, p=0.001, time to reperfusion t=2.708, p=0.009, glucose- NS), and HbA1c was not a predictor of MSI% (R=0.453, R2=0.206, age t=-2.529, p=0.015, time to reperfusion t=-2.237, p=0.030, HbA1c-NS).

## Conclusions

Admission glucose levels are associated with larger infarct size, and HbA1c levels are associated with reduced myocardial salvage. However, glycemic status is not an independent predictor of infarct size or salvage when time to reperfusion is taken in to consideration.

## Funding

Acknowledgements: This work was supported by a British Heart Foundation (BHF) project grant, and the NIHR Cardiovascular Biomedical Research Unit, Leicester, UK.

